# RETRACTION: Paris Saponin VII Enhanced the Sensitivity of HepG2/ADR Cells to ADR via Modulation of PI3K/AKT/MAPK Signaling Pathway

**DOI:** 10.1002/kjm2.70030

**Published:** 2025-04-08

## Abstract

**RETRACTION:** TangG.‐E. , NiuY.‐X. , LiY. , WuC.‐Y. , WangX.‐Y. and ZhangJ. , “Paris Saponin VII Enhanced the Sensitivity of HepG2/ADR Cells to ADR via Modulation of PI3K/AKT/MAPK Signaling Pathway,” The Kaohsiung Journal of Medical Sciences 36, no. 2 (2020): 98–106, 10.1002/kjm2.12145.31688993 PMC11896176

The above article, published online on 05 November 2019 in Wiley Online Library (wileyonlinelibrary.com), has been retracted by agreement between; the journal Editor‐in‐Chief, Wan‐Long Chuang; Kaohsiung Medical University; and John Wiley and Sons Australia. The retraction has been agreed upon due to elements in Figures 4A and 6C, which were found duplicated in other articles published earlier elsewhere, and in some cases representing a different scientific context. The authors did not respond to the concerns raised. The editors have lost confidence in the data presented and consider the conclusions to be substantially compromised. The authors were informed of the retraction but were unavailable for comment.

**RETRACTION:** G.‐E. Tang , Y.‐X. Niu , Y. Li , C.‐Y. Wu , X.‐Y. Wang and J. Zhang , “Paris Saponin VII Enhanced the Sensitivity of HepG2/ADR Cells to ADR via Modulation of PI3K/AKT/MAPK Signaling Pathway,” The Kaohsiung Journal of Medical Sciences 36, no. 2 (2020): 98–106, 10.1002/kjm2.12145.31688993 PMC11896176

The above article, published online on 05 November 2019 in Wiley Online Library (wileyonlinelibrary.com), has been retracted by agreement between; the journal Editor‐in‐Chief, Wan‐Long Chuang; Kaohsiung Medical University; and John Wiley and Sons Australia. The retraction has been agreed upon due to elements in Figures 4A and 6C, which were found duplicated in other articles published earlier elsewhere, and in some cases representing a different scientific context. The authors did not respond to the concerns raised. The editors have lost confidence in the data presented and consider the conclusions to be substantially compromised. The authors were informed of the retraction but were unavailable for comment.

